# Blood Fatty Acid Profile as a Predictor of Antidepressant Efficacy—A Prospective Cohort Pilot Study Protocol

**DOI:** 10.3390/jcm15135081

**Published:** 2026-06-30

**Authors:** Mateusz Kapela, Aleksandra Margulska, Joanna Grzelczyk, Joanna Palma, Grażyna Budryn, Karolina Skonieczna-Żydecka, Ewelina Barszcz, Dominik Strzelecki, Oliwia Gawlik-Kotelnicka

**Affiliations:** 1 Department of Affective and Psychotic Disorders, Medical University of Lodz, Czechoslowacka 8/10, 92-216 Lodz, Poland; mateusz.kapela@stud.umed.lodz.pl (M.K.); dominik.strzelecki@umed.lodz.pl (D.S.); 2 Department of Child and Adolescent Psychiatry, Medical University of Lodz, Czechoslowacka 8/10, 92-216 Lodz, Poland; aleksandra.margulska@umed.lodz.pl; 3 Institute of Food Technology and Analysis, Faculty of Biotechnology and Food Sciences, Lodz University of Technology, Stefanowskiego 2/22, 90-537 Lodz, Poland; joanna.grzelczyk@p.lodz.pl (J.G.); grazyna.budryn@p.lodz.pl (G.B.); 4 Department of Biochemical Science, Pomeranian Medical University in Szczecin, Broniewskiego 24, 71-460 Szczecin, Poland; joanna.palma@pum.edu.pl (J.P.); karolina.skonieczna.zydecka@pum.edu.pl (K.S.-Ż.); 5 Central Teaching Hospital, Medical University of Lodz, Pomorska 251, 92-213 Lodz, Poland; ewelina.barszcz@stud.umed.lodz.pl

**Keywords:** depression, antidepressant treatment response, fatty acids, short-chain fatty acids, gut-brain axis

## Abstract

**Background/Objectives**: Despite the availability of multiple pharmacological treatment options, up to one-third of patients with depressive disorders fail to achieve adequate symptom relief following first-line antidepressant therapy, representing a major unmet clinical need. Fatty acids—including short-chain (SCFAs), medium-chain (MCFAs), and long-chain polyunsaturated fatty acids (PUFAs)—are increasingly implicated in depression pathogenesis through neuroinflammation, gut–brain axis signaling, and neurotransmitter metabolism, but their potential as predictors of antidepressant response remains largely unexplored. The primary aim is to evaluate whether baseline fatty acid profiles can predict pharmacological antidepressant treatment efficacy. The secondary objective is to assess the association between blood fatty acid profile and clinical presentation of depressive disorders. **Methods**: Sixty adults diagnosed with depressive disorders (ICD-11) at the initiation of a new antidepressant treatment will be recruited from psychiatric settings. Fasting blood samples collected at baseline will undergo gas chromatographic analysis of fatty acid methyl esters (FAMEs) and GC-MS quantification of SCFAs (acetic, propionic, and butyric acids). Clinical outcomes will be assessed at baseline and at 3, 6, and 12 weeks using the Beck Depression Inventory-II (BDI-II) and the Depression Anxiety Stress Scales (DASS-42). The primary endpoint is the change in BDI-II total score from baseline to week 6. Treatment response, defined as a ≥50% reduction in BDI-II total score at week 6, and remission, defined as a BDI-II score ≤12 at week 6, will be examined as secondary outcomes. Dietary habits, physical activity, quality of life, and anthropometric parameters will be collected as potential confounders. **Discussion**: This study is among the first prospective investigations to comprehensively characterize the circulating fatty acid spectrum as potential predictors of antidepressant outcomes. Findings may support identification of metabolic phenotypes of depression and contribute to personalized treatment strategies.

## 1. Introduction

### 1.1. Background

Depressive disorders are among the most prevalent mental health conditions worldwide. However, despite the availability of multiple pharmacological treatment options, up to one-third of patients fail to achieve adequate symptom relief following first-line antidepressant therapy, leaving them with persistent symptoms and significantly impaired functioning [1,2]. It causes a tremendous burden, including unemployment, loss of productivity, and increased health care costs. This substantial burden highlights the need for accessible biomarkers that could help identify patients more or less likely to respond to antidepressant treatment [2].

Among polyunsaturated fatty acids (PUFAs), eicosapentaenoic acid (EPA), arachidonic acid (AA), and docosahexaenoic acid (DHA) are of particular interest because of their involvement in neuronal membrane function and lipid-derived signaling. Clinical and observational studies have reported lower circulating EPA concentrations, a reduced EPA, and a relative predominance of DHA in individuals with depressive disorders compared with healthy controls [3,4]. Differences have also been observed in erythrocyte fatty acid composition, including higher n-6 ratios, higher AA relative to EPA and DHA, and lower absolute DHA concentrations. Some cross-sectional and prospective studies further suggest that an elevated AA/(EPA + DHA) ratio is associated with greater depressive symptom severity and increased inflammatory activity [5,6,7].

Evidence from randomized controlled trials and meta-analyses indicates that omega-3 supplementation may produce a modest reduction in depressive symptoms, with more consistent effects reported for EPA-predominant formulations than for DHA-predominant preparations [8,9,10]. Preparations containing at least 60% EPA of the total EPA + DHA content and providing approximately 1–2 g of EPA per day have been associated with the greatest therapeutic benefit [9,10].

Greater effects have also been reported in patients with an inflammatory phenotype, suggesting that inflammatory status may modify the clinical effects of omega-3 fatty acids. These findings provide indirect support for a model in which baseline PUFA composition may be related to treatment outcomes through inflammatory and membrane-related pathways [11,12]. Nevertheless, evidence from supplementation studies does not establish that baseline PUFA concentrations predict response to conventional antidepressant medication.

Short-chain fatty acids (SCFAs), primarily acetate, propionate, and butyrate, are produced by the anaerobic fermentation of dietary fiber by the gut microbiota. After absorption into the systemic circulation, they may influence the gut–brain axis through effects on intestinal and blood–brain barrier integrity, G protein-coupled receptor signaling, immune regulation, and neurotransmitter-related pathways [13,14].

Experimental findings further suggest that SCFAs may affect microglial activation, pro-inflammatory cytokine production, and the expression of neurotrophic factors involved in synaptic plasticity [15,16]. These mechanisms remain incompletely characterized in humans but provide a plausible framework linking altered SCFA availability with depressive symptoms and potentially with treatment responsiveness.

Altered circulating and fecal SCFA profiles have been observed in individuals with major depressive disorder (MDD) compared with healthy controls, and lower concentrations of selected SCFAs have been associated with greater symptom severity [17,18]. In particular, reduced propionic acid concentrations have been reported in individuals with metabolic depression, suggesting that propionate may represent a marker of the metabolic and microbiota-related disturbances present in a subgroup of patients with depression [19]. Although these findings do not demonstrate predictive value, they identify baseline propionic acid as a biologically plausible candidate for further investigation.

Available evidence suggests that depression is also associated with alterations in circulating and membrane concentrations of medium-chain fatty acids (MCFAs). Clinical and metabolomic studies have reported reduced levels of specific MCFAs, such as capric (C10:0) and lauric acid (C12:0), in individuals with depressive symptoms compared to healthy controls. These changes may reflect disturbances in mitochondrial energy metabolism and gut–brain axis signaling [20,21]. However, evidence concerning MCFAs remains limited, and their potential relationship with antidepressant response should be regarded as exploratory.

Taken together, these findings suggest that baseline fatty acid composition may reflect several processes potentially relevant to antidepressant response, including membrane lipid balance, inflammation, gut microbiota-related signaling, and energy metabolism. EPA, DHA, and AA may be particularly relevant to membrane and inflammatory pathways, whereas SCFAs and MCFAs may reflect microbiota-related and metabolic alterations. However, it remains unclear whether baseline fatty acid profiles are associated with subsequent treatment outcomes. Given the limited and heterogeneous evidence concerning individual fatty acids, the present pilot study will examine the full measured fatty acid panel exploratorily rather than test a single prespecified fatty acid hypothesis.

### 1.2. Aims and Hypotheses

Our aims are the following:

- The primary aim of the current pilot study is to investigate the association between baseline fatty acid profiles and the magnitude of antidepressant treatment response and to generate preliminary effect size estimates to inform the design of a future adequately powered study examining the predictive utility of fatty acid biomarkers in depression.
- The secondary objective is to investigate the association between the erythrocyte membrane and serum fatty acid profiles and the clinical presentation of depressive disorders, encompassing the severity of depressive symptoms, anxiety symptoms, and stress-related symptoms, as assessed by the Depression, Anxiety and Stress Scales (DASS-42) and the Beck Depression Inventory-II (BDI-II).

We assume that the profile of long-, medium-, and short-chain fatty acids in blood may be associated with the course and clinical picture of depression. The main confounding factors are hypothesized to be diet, physical activity, and somatic comorbidities, with their treatment.

### 1.3. Outcome Measures

In our study, the primary outcome measure will be the change in depressive symptom severity as measured by the BDI-II total score from baseline to the 6-week assessment, across its three dimensions: cognitive, affective, and somatic.

The effectiveness of antidepressant treatment will be measured by changes in the BDI-II scale. According to previous research, secondary outcome measures will include remission, defined as a score of ≤12, and the minimal clinically important difference, defined as a 17.5% reduction in scores [22].

Treatment response will be operationalized as a ≥50% reduction in BDI-II total score from baseline to the 6-week assessment, consistent with established conventions in antidepressant treatment research, and will also be treated as a secondary outcome measure.

The secondary outcome measures will also include depression, anxiety, and stress symptoms assessed with the DASS-42 subscale scores and quality of life assessed with the WHOQOL-BREF instrument.

## 2. Materials and Methods

### 2.1. Study Design and Population

This protocol was prepared following the Standard Protocol Items: Recommendations for Interventional Trials (SPIRIT) 2025 guidelines, adapted to the extent applicable to the observational prospective design of the present study [23].

Participants will be recruited in the Central Teaching Hospital of the Medical University of Lodz, primary healthcare clinics, and mental health clinics in the Lodz voivodeship. The study population will consist of 60 adults diagnosed with depressive disorders according to the International Classification of Diseases, 11th (ICD-11). Eligible diagnoses will include single-episode depressive disorder, recurrent depressive disorder, and dysthymic disorder, reflecting the spectrum of conditions characterized primarily by depressive symptomatology. Additionally, participants must present with a BDI-II score ≥ 15 at baseline, confirming clinically relevant depressive symptom severity at the time of enrollment.

Each participant will be at the beginning of a new pharmacological antidepressant treatment (5 days before to 5 days after treatment initiation). Antidepressant treatment will not be standardized by the study protocol, as treatment decisions will follow routine clinical practice. Antipsychotic medication used as augmentation of antidepressant treatment will not constitute an exclusion criterion but will be recorded as a combination treatment.

To qualify for the study, participants must meet all inclusion criteria and not meet any exclusion criteria listed in Table 1.

**Figure 1:**
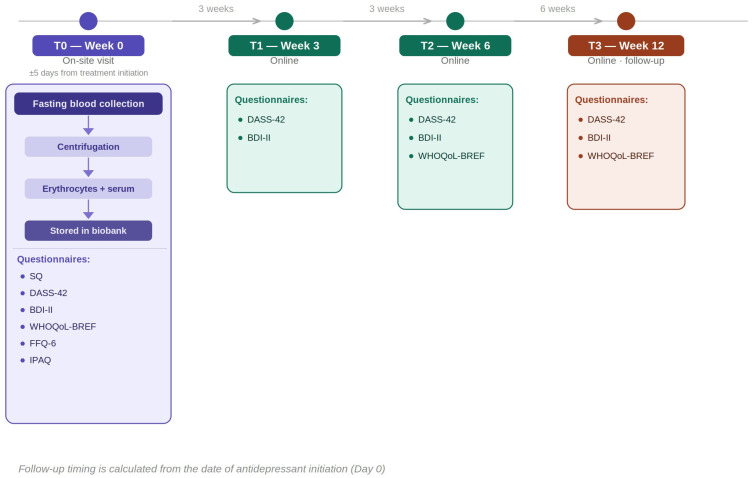
A schematic representation of the study timeline with delineated time points. SQ—study questionnaire, DASS-42—Depression Anxiety Stress Scales, BDI-II—Beck Depression Inventory-II, FFQ-6—Food Frequency Questionnaire-6, IPAQ—International Physical Activity Questionnaire, WHOQoL-BREF—World Health Organization Quality of Life—Brief version.

During the study, each participant will have a scheduled on-site visit, during which questionnaires will be completed, and blood samples will be collected for analysis. After centrifugation, serum aliquots and washed erythrocyte pellets will be frozen and stored under predefined biobanking conditions. Following this visit, three subsequent time points are planned—at 3, 6, and 12 weeks (follow-up)—during which the participant will receive online questionnaires to complete. Follow-up assessments will be scheduled relative to the date of antidepressant treatment initiation. At each follow-up assessment, participants will report current antidepressant treatment, dose changes, discontinuations, augmentation strategies, newly introduced concomitant medications, and psychotherapy participation. Participants will remain in the study if the antidepressant treatment is modified during follow-up. The type, date, and reason for each treatment modification will be recorded. A sensitivity analysis will be performed after excluding participants with major treatment modifications before the week 6 assessment. The time points’ schedule is also illustrated in Figure 1.

### 2.2. Sample Size

As this is a prospective pilot study, the planned sample size was not based on a formal confirmatory power calculation. Currently, there is limited data allowing for a reliable estimation of the expected effect size for the association between baseline fatty acid profiles and antidepressant treatment outcomes. Therefore, the planned recruitment of 60 participants was determined pragmatically, based on the feasibility of recruitment, follow-up, and biological sample processing and on the aim of obtaining preliminary estimates of variability, effect sizes, responder proportions, and attrition rates for a future adequately powered study. Based on previous studies, the anticipated treatment response rate is approximately 50% [24]. Therefore, before accounting for attrition, the planned sample of 60 participants may yield approximately 30 responders and 30 non-responders. This estimate is provided to describe the expected sample distribution and does not imply that the study is adequately powered to develop or validate a multivariable prediction model [25].

### 2.3. Scales and Questionnaires

A study questionnaire (SQ) will be used to collect basic socioeconomic data as well as various health-related information. Participants will be asked to provide, among other things, personal details, place of residence, and occupation, as well as information on dietary supplement use, current medications, smoking habits, chronic diseases, and psychotherapy. Use of medications or supplements potentially affecting lipid metabolism, inflammatory status, or gut microbiota composition, including statins, corticosteroids, antibiotics, probiotics, and omega-3 supplements, will also be recorded. For participants receiving antidepressant treatment, the SQ will also record medication class (SSRI, SNRI, or others), dose expressed as fluoxetine equivalents, and use of combination therapy. As most participants will be inpatients at baseline, adherence to antidepressant pharmacotherapy is expected to be high during the initial phase of treatment and will be monitored as part of routine clinical care. Following discharge, participants will be provided with a structured medication adherence form, on which they will be asked to record each administered dose on a daily basis. Completed forms will be collected and reviewed at each subsequent follow-up time point. These variables will be described and, depending on their frequency and data distribution, considered as potential confounders in analyses of treatment response.

Beck Depression Inventory-II (BDI-II) is a widely used self-report questionnaire designed to assess the severity of depressive symptoms in adolescents and adults. The instrument consists of 21 items, each reflecting a specific symptom or attitude related to depression, in accordance with diagnostic criteria for depressive disorders. Respondents are asked to rate how they have been feeling over the preceding two weeks, including the day of assessment. Each item is scored on a 4-point scale ranging from 0 to 3, with higher total scores indicating greater severity of depressive symptoms. The total score ranges from 0 to 63 and is interpreted using established cut-off values: 0–13 indicating minimal, 14–19 mild, 20–28 moderate, and 29–63 severe depressive symptoms [26].

The BDI-II captures three key dimensions of depression: the cognitive dimension, reflecting negative thoughts, pessimism, and self-evaluation; the affective dimension, encompassing mood disturbances such as sadness and loss of pleasure; and the somatic dimension, addressing physical symptoms including changes in sleep, appetite, energy levels, and psychomotor functioning [27].

The Polish version of the Beck Depression Inventory-II (BDI-II), which has been psychometrically validated for internal consistency, construct validity, and criterion validity in a Polish population, will be used to assess depressive symptom severity [28].

Depression, Anxiety, Stress Scales (DASS-42) is a self-report instrument designed to assess the severity of symptoms related to depression, anxiety, and stress in adults. The scale consists of 42 items, divided into three subscales, each comprising 14 items: Depression, Anxiety, and Stress [29].

The Depression subscale assesses dysphoria, hopelessness, lack of interest, self-deprecation, and anhedonia. The Anxiety subscale focuses on autonomic arousal, skeletal muscle effects, situational anxiety, and subjective experiences of anxious affect. The Stress subscale measures chronic non-specific arousal, including difficulty relaxing, irritability, nervous tension, and agitation.

The Polish validated version of the Depression Anxiety Stress Scales (DASS-42), developed by Makara-Studzińska et al., will be used in this study [30].

WHO Quality of Life (WHOQOL-BREF) is a standardized self-report questionnaire developed by the World Health Organization to assess subjective quality of life in adults. It is a shortened version of the WHOQOL-100 and consists of 26 items. The instrument measures quality of life across four domains: physical health, psychological health, social relationships, and environment, as well as two general items assessing overall quality of life and general health perception. Participants rate each item on a 5-point Likert scale, reflecting intensity, capacity, frequency, or evaluation. Domain scores are calculated according to WHO guidelines, with higher scores indicating better perceived quality of life [31]. The study will use the Polish validated version of the WHOQOL-BREF questionnaire by Jaracz et al. [32].

The Frequency Questionnaire (FFQ-6) is a dietary assessment tool designed to evaluate habitual food consumption patterns over a specified retrospective period. The questionnaire focuses on the frequency of intake of selected food groups. Participants are asked to report how often they consume specific food items or food categories using predefined frequency options. The FFQ-6 enables estimation of dietary patterns, nutrient intake proxies, and adherence to specific dietary behaviors, including those relevant to lipid and fatty acid intake. The study will use the Polish validated version of the FFQ-6 questionnaire by Niedzwiedzka et al. [33].

Dietary data obtained from the FFQ-6 will be aggregated into predefined food groups for the purpose of statistical adjustment. The following groups will be distinguished: sweets and snacks, dairy and eggs, cereal products, oils, fruits, vegetables and seeds, meat (including fish and its subcategories of processed and unprocessed meat), alcohol, and drinks excluding water. Selected groups will be further subdivided into processed and unprocessed subcategories. For each group, a dietary index will be calculated based on the mean consumption frequency of constituent products. A composite processed food index will additionally be derived from items classified as processed across multiple food categories. These indices will serve as dietary covariates in regression analyses examining fatty acid profiles in relation to clinical outcomes [34].

The International Physical Activity Questionnaire (IPAQ) was developed by an international consortium to provide a standardized measure of physical activity suitable for international and cross-cultural use. A large reliability and validity study conducted across 12 countries (including translation and cultural adaptation procedures) showed acceptable measurement properties [35]. The study will use the Polish version of the IPAQ compiled by Biernat et al., which has been officially translated and registered for use in Poland and enables classification of physical activity into internationally standardized MET-minute categories [36].

### 2.4. Biological Parameters

Physical examination parameters: weight, body mass index (BMI), and waist circumference (WC).

Anthropometric indicators such as BMI and waist circumference are essential covariates in research examining circulating fatty acid levels.

BMI provides a general estimate of overall adiposity, which influences lipid metabolism, fatty acid storage, and mobilization. Individuals with higher adiposity often present altered profiles of circulating fatty acids due to changes in lipid turnover, insulin sensitivity, and adipose tissue function. Waist circumference, in contrast, is a specific marker of visceral fat accumulation, which is metabolically more active than subcutaneous fat.

Visceral adiposity is strongly associated with disruptions in lipid homeostasis, increased lipolysis, and elevated flux of free fatty acids into the bloodstream. Consequently, it may significantly modify the concentrations of fatty acids measured in plasma or serum [37].

Blood samples will be collected in the morning after an overnight fast by qualified medical personnel. CRP and complete blood count will be assessed in all participants using blood collected during the baseline visit. The neutrophil-to-lymphocyte ratio will be calculated as the absolute neutrophil count divided by the absolute lymphocyte count.

To obtain serum, blood will be collected into tubes without anticoagulants or preservatives and kept in an upright position at room temperature for 30–45 min to allow for clot formation. The samples will then be centrifuged for approximately 10–15 min at 3000× *g*. After centrifugation, the serum will be carefully aspirated and transferred into patient code-labeled cryovials.

To obtain the erythrocyte pellet for FAME analysis, blood will be collected into tubes containing ethylenediaminetetraacetic acid (EDTA) and gently mixed immediately after collection. The samples will then be centrifuged for approximately 10–15 min at 3000× *g*. After centrifugation, the plasma will be carefully aspirated in order to isolate the pellet which will be washed twice with isotonic saline to remove residual proteins and transferred into patient code-labeled cryovials.

Post-centrifugation pellet samples will be frozen and stored in the Biobank.

The samples will be transported on dry ice from the Medical University of Lodz to the collaborating laboratories.

Samples will be identified exclusively by study codes, and laboratory personnel will not have access to personal identifiers or treatment outcome data.

Blood will be collected to determine the profile of short-chain fatty acids in serum as well as the erythrocyte membrane medium- and long-chain fatty acid composition. The simultaneous assessment of erythrocyte membrane fatty acids and serum short-chain fatty acids reflects the complementary and mechanistically distinct roles these lipid fractions play in depression pathophysiology. Erythrocyte membrane fatty acid composition—particularly the balance of n-6 and n-3 polyunsaturated fatty acids—provides an integrated index of long-term fatty acid status and membrane-level inflammatory potential, reflecting dietary intake and metabolic processing over the preceding 90–120 days [38]. In contrast, serum SCFAs represent dynamic, rapidly fluctuating metabolites produced by gut microbiota fermentation, serving as proxies of gut microbial activity and gut–brain axis signaling at the time of assessment [39]. By capturing both long-term structural fatty acid status and acute microbial metabolite levels within the same participants, this study aims to provide a comprehensive and mechanistically grounded characterization of the lipid-related biological landscape associated with depressive disorders and antidepressant treatment response.

Certified reference standards of saturated, monounsaturated, and polyunsaturated fatty acids ranging from C10 to C24 will be used, including decanoic (C10:0), undecanoic (C11:0), lauric (C12:0), myristic (C14:0), myristoleic (C14:1), pentadecanoic (C15:0), palmitic (C16:0), palmitoleic (C16:1), margaric (C17:0), heptadecenoic (C17:1), stearic (C18:0), vaccenic (C18:1n7), oleic (C18:1n9), linoleic (C18:2n6), γ-linolenic (C18:3n6), α-linolenic (C18:3n3), arachidic (C20:0), eicosenoic (C20:1), eicosatrienoic (C20:3n6), arachidonic (C20:4n6), eicosapentaenoic (C20:5n3), behenic (C22:0), erucic (C22:1n9), docosatetraenoic (C22:4n6), docosapentaenoic (C22:5n3), docosahexaenoic (C22:6n3), tricosanoic (C23:0), lignoceric (C24:0), and nervonic (C24:1) acids. Heneicosanoic acid (C21:0) will be used as an internal standard.

FAMEs will be extracted from the erythrocyte pellet using a modified Folch procedure. Samples will be mixed with chloroform and methanol (2:1, *v*/*v*), followed by the addition of the internal standard and butylated hydroxytoluene. The mixtures will be agitated for 20 min and centrifuged, after which the supernatant will undergo saponification with potassium hydroxide in methanol at approximately 70 °C, followed by methylation using boron trifluoride in methanol under the same conditions. After derivatization, FAMEs will be extracted into hexane following the addition of saturated sodium chloride solution and transferred to chromatographic vials.

Gas chromatographic analysis will be performed using an Agilent 7890A system (Agilent Technologies, Santa Clara, CA, USA) equipped with a Supelcowax™ 10 capillary column. A defined oven temperature program will be applied, with hydrogen used as the carrier gas at a constant flow rate. Fatty acids will be identified by comparison of retention times with authentic standards, and quantitative analysis will be conducted using the ChemStation LTS 01.11 software, with relative fatty acid concentrations calculated in reference to the internal standard. The analytical procedure will follow established lipidomic methodologies.

Serum concentrations of acetate, propionate, and butyrate will be quantified using gas chromatography–mass spectrometry at the Institute of Food Technology and Analysis, Lodz University of Technology. These three SCFAs were selected as they represent the most abundant products of anaerobic fermentation of dietary fiber by the gut microbiota, collectively accounting for over 90% of total SCFA production in the human colon, and have been most consistently implicated in gut–brain axis signaling and neuroinflammatory modulation relevant to depressive disorders.

The analysis of SCFAs will be performed on a GCMS-2010 (Shimadzu Corporation, Kyoto, Japan). A split/splitless injector will be set to 200 °C. The temperature program will be as follows: 100 °C (3 min hold) ramped to 210 °C (0.5 min hold) at 30.0 °C min^−1^. Helium will be used as the carrier gas at a constant linear velocity of 50 cm s^−1^ (with an initial inlet pressure of 133.0 kPa). The injection volume will be 8.0 μL with a split ratio of 1:5.

The MS system will operate in electron ionization (EI) mode using an ionization energy of 70 eV, and it will acquire the target compounds in selected ion monitoring (SIM) mode, monitoring three different fragment ions for each compound within specific time acquisition windows. The following ions will be selected: propionic acid, 74 *m*/*z* (Q); acetic acid, 60 *m*/*z* (Q); and butyric acid, 88 *m*/*z* (Q). MS parameters will be set as follows: an ion source temperature of 220 °C and interface temperature of 220 °C. The GCMS solution software 4.45 will be used for both data acquisition and processing.

To ensure the quality of the analyses, four replicates will be performed. The scan mass range will be 30–400 *m*/*z*. Compound identification will be performed using commercial standard kits. Prior to analysis, a system suitability test will be performed by injection. Five replicates will be performed using a 0.40 mg/mL standard solution. The percentage relative standard deviation (%RSD) of retention time (RT) and peak area will be calculated using acceptance criteria of RSD ≤ 1% and RSD ≤ 2.5%, respectively.

### 2.5. Ethics

The study will be carried out in accordance with the principles of the Declaration of Helsinki. Approval for the entire study protocol was obtained by the principal study investigator (PSI) from the Bioethics Committee of the Medical University of Lodz (Bioethics Committee resolution no. RNN/274/25/KE, dated 18 November 2025).

All participants will be provided with written information detailing the study objectives and procedures. Enrollment will occur only after eligibility criteria are met and written informed consent is obtained. Participants will be informed of their right to withdraw from the study at any time without any adverse consequences. Participation in the study involves minimal risk, mainly related to blood sampling such as transient discomfort, bruising, or rarely fainting.

### 2.6. Statistical Analysis

All analyses will be exploratory and hypothesis-generating. Continuous variables will be summarized using means and standard deviations or medians and interquartile ranges, as appropriate, and categorical variables as counts and percentages. Markedly skewed fatty acid variables may be transformed. Fatty acid measures will be standardized to facilitate comparison of effect estimates. Results will be reported with 95% confidence intervals, and *p*-values will be interpreted in the context of the pilot nature of the study. The primary endpoint will be the change in BDI-II total score from baseline to week 6. Associations between individual baseline fatty acids and depressive symptom trajectories will be examined using separate linear mixed-effects models estimated with restricted maximum likelihood. Repeated BDI-II scores obtained at baseline and at weeks 3, 6, and 12 will constitute the dependent variable. Models will include time, the standardized fatty acid measure, the fatty acid-by-time interaction, age, sex, and antidepressant class as fixed effects, with a participant-specific random intercept. The prespecified primary contrast will represent the association between the baseline fatty acid measure and change in BDI-II score from baseline to week 6. Fatty acids will be analyzed individually rather than entered simultaneously into a single conventional multivariable model.

Antidepressant class will initially be categorized as selective serotonin reuptake inhibitor, serotonin–norepinephrine reuptake inhibitor, or other. Categories may be combined if their sizes do not permit reliable estimation. The robustness of the primary findings will be examined in sensitivity models additionally accounting for relevant dietary, metabolic, lifestyle, and treatment-related factors. These may include fish and seafood intake, a plant- and fiber-rich food index, body mass index or waist circumference, physical activity, smoking, alcohol consumption, comorbidities, concomitant medications, psychotherapy, adherence, hospitalization status, and treatment modifications. Because of the limited sample size, these variables will not all be included simultaneously in a single model. Secondary binary outcomes at week 6 will include treatment response, remission, and minimal clinically important improvement. Their associations with individual fatty acids will be examined using separate exploratory logistic regression models with a limited number of prespecified covariates. Penalized likelihood estimation may be used in the event of complete or quasi-complete separation. 

Longitudinal changes in DASS-42 subscales and WHOQOL-BREF domain scores will be examined using analogous mixed-effects models. Baseline relationships between fatty acid measures and psychometric variables will be assessed using Pearson or Spearman correlation coefficients, as appropriate. Principal component analysis will be applied to standardized erythrocyte fatty acid variables to identify broader fatty acid patterns and reduce dimensionality. The number of retained components will be determined using parallel analysis and inspection of the scree plot. Component scores will be evaluated in exploratory models analogous to those used for individual fatty acids. PCA will not be applied to the three serum SCFAs. The Benjamini–Hochberg false discovery rate procedure will be used to account for multiple testing involving individual fatty acids. Correction will be applied separately to the primary family of analyses involving the week 6 BDI-II outcome and to each secondary outcome family. Both unadjusted *p*-values and false-discovery-rate-adjusted *q*-values will be reported. Missing follow-up data will be minimized through regular contact with participants and electronic questionnaires requiring completion of all items. The timing and reasons for missed assessments and withdrawal will be recorded. Mixed-effects models will use all available repeated observations and will provide valid estimates under the missing-at-random assumption. Missingness patterns will be examined descriptively, and the potential impact of departures from this assumption will be evaluated in sensitivity analyses if the amount of missing data permits. Missing baseline fatty acid measurements will not be imputed. The study is not designed to establish clinical cut-offs or validate a clinical prediction tool. If an exploratory combined prediction model is developed, its apparent performance and optimism will be assessed using bootstrap resampling. Such findings will require external validation in future adequately powered studies.

Analyses will be performed using Statistica 13.3 and the R software 4.6.1.

## 3. Discussion

As highlighted earlier, the rising prevalence of depressive disorders has intensified the need for effective predictors of antidepressant treatment response. This growing clinical challenge is reflected in the increasing scientific interest in identifying reliable biomarkers of treatment efficacy, which has led to a substantial expansion of research in this area. The studies summarized in Table 2 illustrate the diversity of approaches and findings to date, underscoring both the potential and the current limitations of existing predictors of antidepressant treatment outcomes.

Compared with previous studies focusing primarily on selected inflammatory markers or individual lipid fractions, the present prospective pilot study is distinguished by its comprehensive assessment of circulating fatty acids, including short-, medium-, and long-chain fatty acids. Importantly, the inclusion of short-chain fatty acids, which are closely linked to gut microbiota metabolism, represents a novel approach in the context of antidepressant treatment response. The prospective design and 12-week follow-up allow for evaluation of fatty acids as potential predictors rather than correlates of clinical response, while the naturalistic treatment setting enhances the clinical relevance of the findings.

While the current pilot study is not powered to define definitive clinical diagnostic thresholds, it serves as a critical translational stepping stone. The primary objective is to estimate the effect sizes and variance of fatty acid parameters in relation to antidepressant response, and these estimates will allow for precise a priori sample size calculations for subsequent large-scale, multi-center cohorts designed specifically to establish predictive performance metrics, including diagnostic thresholds derived from ROC-based analyses with associated sensitivity, specificity, and positive predictive values.

Regarding personalized antidepressant strategies, we hypothesize that fatty acid profiling could eventually facilitate a ‘match/mismatch’ clinical approach. Identifying a patient with a low baseline EPA:DHA ratio, reduced erythrocyte EPA and DHA levels, an elevated AA/(EPA + DHA) ratio, or depleted SCFA concentrations—potentially in combination with elevated inflammatory markers such as CRP or NLR, and indicators of gut microbiota dysregulation—may indicate an underlying inflammatory or gut-dysbiosis-associated phenotype of depression. In clinical practice, such a biomarker profile could flag a patient likely to respond poorly to a standard monotherapy but who might benefit from targeted adjunctive interventions—such as nutritional or anti-inflammatory strategies—tailored to their metabolic phenotype. Furthermore, by adjusting for dietary intake assessed with the FFQ-6 and anthropometric parameters, this study will provide foundational data on whether fatty acid thresholds need to be personalized based on metabolic characteristics, bringing precision psychiatry closer to routine clinical implementation.

Several limitations of the present study should be acknowledged. First, depressive symptom severity and treatment response will be assessed exclusively using self-reported measures, namely the BDI-II and DASS-42, without the inclusion of clinician-rated scales such as the Hamilton Depression Rating Scale (HAM-D) or the Montgomery–Åsberg Depression Rating Scale (MADRS). This should be considered when interpreting treatment response and remission outcomes.

Second, the study does not include a healthy control group. While the primary objective is to identify predictors of antidepressant treatment response rather than to characterize fatty acid abnormalities relative to a reference population, the absence of controls limits the ability to contextualize baseline fatty acid profiles and to draw conclusions regarding disease-specific alterations in lipid metabolism.

Third, participants will be recruited exclusively from clinical settings. While this approach ensures diagnostic rigor and clinical homogeneity of the study sample, it may limit the generalizability of findings to individuals with depressive disorders who do not seek specialist care or who are managed outside of institutional healthcare settings.

## Figures and Tables

**Table 1:** Eligibility criteria.

Inclusion Criteria	Exclusion Criteria	Reasons for the Participant to Be Discontinued from the Study
1. Age ≥ 18 years	1. Pregnancy	1. Withdrawal of informed consent to participate in the study
2. Diagnosis of depressive disorder according to ICD-11 and baseline BDI-II score ≥ 15 at the time of enrollment	2. Current psychotic symptoms or a primary psychotic disorder	2. Occurrence of any of the study’s exclusion criteria
3. Beginning of a new pharmacological antidepressant treatment, defined as within ±5 days from treatment initiation	3. Depressive symptoms secondary to substance use disorders or organic disorders	
4. Ability to understand the study procedures and provide written informed consent		

**Table 2:** Selected studies investigating biological and metabolic predictors of antidepressant treatment response.

Study	Study Design	Sample Size	Type of Predictors Assessed	Follow-up Duration	Findings
Gut microbiome: A potential indicator for predicting treatment outcomes in major depressive disorder [40]	Prospective cohort study	63 patients with MDD	Gut microbiome diversity and OTUs	8 weeks	Baseline gut microbiota and lipid-related metabolites differed between responders and non-responders; a total of 20 differential metabolites between responders and non-responders that were primarily involved in lipid metabolism
Omega-3 fatty acids for inflamed depression—A match/mismatch study [11]	Randomized controlled trial	101 patients with MDD	Inflammatory status (high vs. low CRP) + EPA supplementation	12 weeks	Participants with high CRP showed greater improvement with EPA augmentation than those with low inflammation
Inflammation as a predictive biomarker for response to omega-3 fatty acids in major depressive disorder: a proof-of-concept study [41]	Randomized controlled trial	68	Inflammatory markers (CRP, IL-6, adiponectin, and IL-1ra) + EPA supplementation	8 weeks	Higher baseline inflammatory markers were associated with improved antidepressant outcomes with EPA supplementation compared with placebo
Biomarkers for response in major depression: comparing paroxetine and venlafaxine from two randomized placebo-controlled clinical studies [42]	Randomized placebo-controlled clinical studies	210	IL-6, IL-10, TNF-α, and CRP	10 weeks	Baseline cytokines predicted paroxetine response; CRP predicted venlafaxine response

MDD—Major depressive disorder; OTUs—Operational taxonomic units; CRP—C-reactive protein; EPA—Eicosapentaenoic acid; IL-6—Interleukin-6; IL-10—Interleukin-10; TNF-α—Tumor necrosis factor-α.

## Data Availability

No new data were created or analyzed in this study.

## Abbreviations

The following abbreviations are used in this manuscript:

AA	Arachidonic acid
BDI-II	Beck Depression Inventory-II
BMI	Body mass index
BDNF	Brain-derived neurotrophic factor
CRP	C-reactive protein
DASS-42	Depression Anxiety Stress Scale
DHA	Docosahexaenoic acid
EDTA	Ethylenediaminetetraacetic acid
EPA	Eicosapentaenoic acid
FAMEs	Fatty acid methyl esters
FDR	False discovery rate
FFQ-6	Food Frequency Questionnaire
GABA	Gamma-aminobutyric acid
GC-MS	Gas chromatography–mass spectrometry
ICD-11	International Classification of Diseases, 11th revision
IPAQ	International Physical Activity Questionnaire
MCFAs	Medium-chain fatty acids
MDD	Major depressive disorder
MET	Metabolic equivalent of task
MUFAs	Monounsaturated fatty acids
NLR	Neutrophil-to-lymphocyte ratio
OTUs	Operational taxonomic units
PUFAs	Polyunsaturated fatty acids
SCFAs	Short-chain fatty acids
SFAs	Saturated fatty acids
SNRI	Serotonin–norepinephrine reuptake inhibitor
SSRI	Selective serotonin reuptake inhibitors
SQ	Study questionnaire
TNF-α	Tumor Necrosis Factor-alpha
WC	Waist circumference
WHOQOL-BREF	World Health Organization Quality of Life—Brief version
